# Floral scent and species divergence in a pair of sexually deceptive orchids

**DOI:** 10.1002/ece3.3147

**Published:** 2017-06-28

**Authors:** Daniel D. L. Gervasi, Marc‐Andre Selosse, Mathieu Sauve, Wittko Francke, Nicolas J. Vereecken, Salvatore Cozzolino, Florian P. Schiestl

**Affiliations:** ^1^ Department of Systematic and Evolutionary Botany University of Zürich Zürich Switzerland; ^2^ Institut de Systématique, Évolution, Biodiversité (ISYEB) UMR 7205 CNRS MNHN UPMC EPHE Muséum national d'Histoire naturelle Sorbonne Universités Paris France; ^3^ Department of Plant Taxonomy and Nature Conservation University of Gdansk Gdańsk Poland; ^4^ Institute of Organic Chemistry University of Hamburg Hamburg Germany; ^5^ Agroecology and Pollination Group Landscape Ecology and Plant Production Systems Université libre de Bruxelles (ULB) Brussels Belgium; ^6^ Department of Biology University of Naples Federico II Naples Italy

**Keywords:** adaptation, density‐dependent selection, floral volatiles, mycorrhizal fungi, *Ophrys aymoninii*, *Ophrys insectifera*, pollinator switch, sexual deception

## Abstract

Speciation is typically accompanied by the formation of isolation barriers between lineages. Commonly, reproductive barriers are separated into pre‐ and post‐zygotic mechanisms that can evolve with different speed. In this study, we measured the strength of different reproductive barriers in two closely related, sympatric orchids of the *Ophrys insectifera* group, namely *Ophrys insectifera* and *Ophrys aymoninii* to infer possible mechanisms of speciation. We quantified pre‐ and post‐pollination barriers through observation of pollen flow, by performing artificial inter‐ and intraspecific crosses and analyzing scent bouquets. Additionally, we investigated differences in mycorrhizal fungi as a potential extrinsic factor of post‐zygotic isolation. Our results show that floral isolation mediated by the attraction of different pollinators acts apparently as the sole reproductive barrier between the two orchid species, with later‐acting intrinsic barriers seemingly absent. Also, the two orchids share most of their fungal mycorrhizal partners in sympatry, suggesting little or no importance of mycorrhizal symbiosis in reproductive isolation. Key traits underlying floral isolation were two alkenes and wax ester, present predominantly in the floral scent of *O. aymoninii*. These compounds, when applied to flowers of *O. insectifera*, triggered attraction and a copulation attempt of the bee pollinator of *O. aymoninii* and thus led to the (partial) breakdown of floral isolation. Based on our results, we suggest that adaptation to different pollinators, mediated by floral scent, underlies species isolation in this plant group. Pollinator switches may be promoted by low pollination success of individuals in dense patches of plants, an assumption that we also confirmed in our study.

## 1 INTRODUCTION

In the last decades, an increasing number of studies have focused on the strength and evolution of reproductive isolating barriers among co‐occurring species (Coyne & Orr, 1989, 2004; Ramsey, Bradshaw, & Schemske, 2003; Schemske, 2010; Scopece, Widmer, & Cozzolino, 2008). Depending on the timing of their onset, reproductive isolation barriers are classified as either pre‐zygotic (e.g., behavioral, mechanical, or gametic isolation) or post‐zygotic (e.g., hybrid sterility or ecological inviability) (Coyne & Orr, 2004). The local maintenance of distinct species usually requires a combination of different types of barriers, and their hierarchical importance is often taxon‐specific (Coyne & Orr, 1998; Rieseberg & Willis, 2007).

To highlight the role of reproductive isolation in speciation, reproductive barriers must be investigated in species that diverged recently (Coyne & Orr, 2004). In addition, the sequence of evolution of different barriers can be informative of their relative importance during speciation. In plants, pre‐zygotic barriers (e.g., floral isolation) often predate post‐zygotic barriers, and are, therefore, thought to play a more critical role during speciation (Coyne & Orr, 2004; Grant, 1994; Kirkpatrick & Ravigne, 2002; Lowry, Modliszewski, Wright, Wu, & Willis, 2008; Moyle, Olson, & Tiffin, 2004; Rieseberg & Willis, 2007; Widmer, Lexer, & Cozzolino, 2009). An example for this are plant adaptations to different pollinators, with reduced gene flow between individuals attracting different pollen vectors (Kay, 2006; Ramsey et al., 2003; Sun, Schlüter, Gross, & Schiestl, 2015; Van der Niet, Peakall, & Johnson, 2014; Waelti, Muhlemann, Widmer, & Schiestl, 2008; Widmer et al., 2009). Such switches in pollinators are thought to be driven by spatially heterogeneous distributions of pollinators, the so‐called pollinator mosaic (Gervasi & Schiestl, 2017; Johnson, 2006; Van der Niet, Pirie, Shuttleworth, Johnson, & Midgley, 2014), or competition for access to pollinators in large plant populations (Waser & Campbell, 2004). Such competition can take the form of a negative association between pollination success and density or population size of conspecifics using the same kinds of pollinators. This is expected to be a more common situation in deceptive plants, where pollen limitation is often severe and pollinators learn to avoid plants after unsuccessful visits (Fritz & Nilsson, 1994; Johnson & Schiestl, 2016).

A plant group where floral isolation may play an especially important role are the orchids, and even more so the sexually deceptive orchids (Ayasse, Stökl, & Francke, 2011; Peakall et al., 2010; Schiestl & Schlüter, 2009; Sedeek et al., 2014; Whitehead & Peakall, 2014; Xu et al., 2011). Sexual deception, currently known in several genera of orchids, and one genus of Asteraceae and Iridaceae, respectively, is a highly specific pollination mechanism (Gaskett, 2011; Johnson & Schiestl, 2016). Sexually deceptive plants mimic mating signals of their pollinators, such as sex pheromones, morphology, and surface pilosity, and entice their pollinators into attempted copulations with their flowers (Peakall & Whitehead, 2014; Peakall et al., 2010; Schiestl & Schlüter, 2009; Schiestl et al., 1999). In this form of floral mimicry, floral odor usually plays a key role in attracting pollinators (Mant, Peakall, & Schiestl, 2005; Schiestl et al., 1999; Sedeek et al., 2014). In the European orchid genus *Ophrys,* pollinator‐attracting scent consists of a blend of cuticular hydrocarbons (alkanes, alkenes (Schlüter & Schiestl, 2008; Ayasse et al., 2011; Xu, Schlüter, & Schiestl, 2012; Xu, Schlüter, Grossniklaus, & Schiestl, 2012), but more polar compounds such as esters may also be important (Gögler et al., 2009).

While the emphasis in sexually deceptive orchids has been on pollinator attraction and its role in reproductive isolation, little is known about the effects of mycorrhizal fungi on reproductive isolation and speciation (Roche et al., 2010). For germination, orchids strongly depend on soil fungi. Their small seeds lack starch reserves and only germinate upon colonization by a soil fungus (Dearnaley, Perotto, & Selosse, 2016) that provides them with nutrients supporting germination until they eventually become photosynthetic. Orchids often depend on specific fungi (e.g., Tulasnellaceae or Serendipitaceae), collectively called rhizoctonias (Dearnaley, Martos, & Selosse, 2013). Recent studies have hypothesized that associations to specific mycorrhizal fungi may act as an extrinsic post‐zygotic barrier by preventing the germination of hybrid seeds through the lack of a proper fungal partner (Jacquemyn, Brys, Cammue, Honnay, & Lievens, 2011; Scopece et al., 2008). Changes in mycorrhizal fungi have thus the potential to drive orchid speciation (Bateman et al., 2014; Otero & Flanagan, 2006; Waterman & Bidartondo, 2008), and there is evidence, although limited, that the sharing of similar fungi is prerequisite for successful establishment of hybrids in orchids (Schatz et al., 2010).

In this study, we investigated multiple potential reproductive barriers and inferred mechanisms of diversification in two species of the *Ophrys insectifera* group. We focused on the following specific questions: (1) Which reproductive barriers maintain species boundaries? (2) Which plant traits underlie reproductive isolation? (3) Is population density negatively associated with fecundity?

## 2 MATERIALS AND METHODS

### 2.1 Study species and site

**Figure 1:**
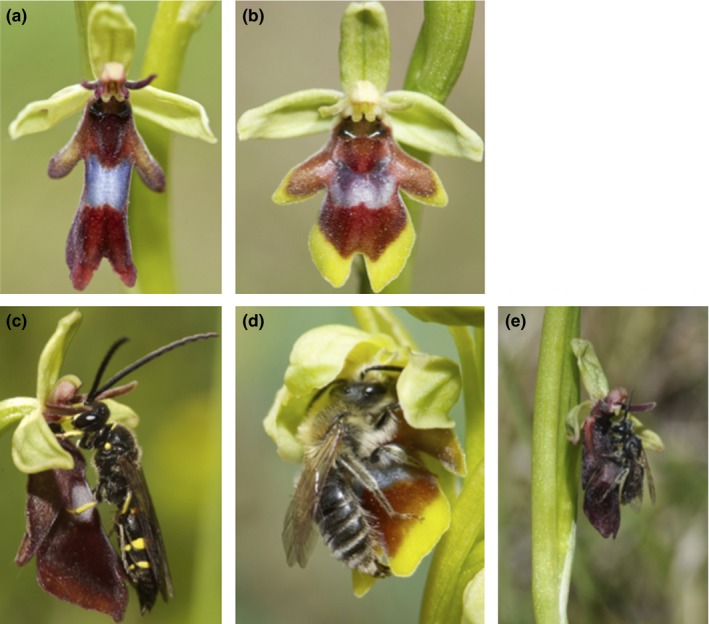
Pictures of the species investigated in this study. Flowers of (a) *Ophrys insectifera,* and (b) *Ophrys aymoninii*. Flowers with pollinators, (c) *O. insectifera* with male digger wasp, *Argogorytes mystaceus,* and (d) *O. aymoninii* with male *Andrena combinata* bee. (e) Scent‐manipulated *O. insectifera* with the “wrong” pollinator, a male *Andrena combinata* bee

Within the sexually deceptive orchids, the *Ophrys insectifera* group offers a unique system for investigating reproductive barriers between groups with different pollinators. The monophyletic *O. insectifera* group consists of three species, namely *O. insectifera* (Figure 1a), *O. subinsectifera*, and *O. aymoninii* (Figure 1b) (Breitkopf, Onstein, Cafasso, Schlüter, & Cozzolino, 2015; Devey, Bateman, Fay, & Hawkins, 2008). *Ophrys insectifera* has a wide distribution and is pollinated by males of two digger wasp species (*Argogorytes mystaceus* and *A. fargeii;* Figure 1c) (Delforge, 2005; Kullenberg, 1951). *Ophrys aymoninii* is a narrow endemic found in the southern Massif Central in France and pollinated by males of the solitary bee *Andrena combinata* (Figure 1d). It regularly occurs in sympatry with the geographically widespread *O. insectifera*. A time‐calibrated maximum clade credibility tree (Breitkopf et al., 2015) supports a very recent divergence between *Ophrys insectifera* and *Ophrys aymoninii* (i.e., in the last 500,000 years). Our study was performed in the Parc Naturel Régional des Grands‐Causses in Aveyron, France during May/June 2010–2013 where the two species flower simultaneously. In total, seven populations were studied in these 4 years (Table S1). For a better visualization of the sympatric occurrence, we collected GPS points of randomly selected plants of both species in the mixed populations (Fig. S1).

### 2.2 Pre‐pollination pre‐zygotic isolation: floral isolation (RI_floral_)

In our study, we mainly focused on ethological floral isolation. Morphologic isolation does occur in *Ophrys*, namely through positioning of pollinia on either the head or abdomen of a pollinator, but it is often not sufficiently precise to prevent cross‐pollination (Cortis et al., 2009; Vereecken, Cozzolino, & Schiestl, 2010). In the here investigated *Ophrys* species, pollinia are deposited on the head of the pollinator (Figure 1). Differences in flower size between the species (Triponez et al., 2013) may still contribute to morphologic isolation between *O. aymoninii* and *O. insectifera*, and they were, however, not quantified in this study. Ethological floral isolation, measured as the ratio between intra‐ and interspecific pollination events, was estimated by tracking the transfer of stained pollinia within/between *O. aymoninii* and *O. insectifera* flowers. To do so, picked *O. aymoninii* and *O. insectifera* plants were set up in plots of four plants along transects with 5–11 plots through the orchids’ habitats (in total 318 plants were used). Plants of both species were collected at the locations Avey2–Avey8 (Table S1). Every plot contained two *Ophrys* plants from each species, set up in 15‐ml falcon tubes filled with water, and positioned in a square (0.2 m distance to the next plant). The pollinia of each species were stained with a distinct color using histologic stains: 2% (weight/volume) Trypan red (72210‐25G, Sigma‐Aldrich) for *O. insectifera* and 1% (w/v) brilliant green (B6756‐100G, Sigma‐Aldrich) for *O. aymoninii*, as described in Xu et al. (2011). The distance between each plot in a transect was 20 m. The plants were examined every 5 days for pollinia removal as well as deposition of stained massulae (pollen packages) on stigmata. Floral isolation (RI_floral_) was calculated as 1 − (total number of interspecific pollination events/total number of intraspecific pollination events) (Scopece, Musacchio, Widmer, & Cozzolino, 2007). This value can vary between 0 (no floral isolation) and 1 (total floral isolation). In 2010, two experimental transects were performed at the location Avey3 and one in Avey2. In 2011, one experimental transect was performed at the locations Avey2 and Avey3. In 2012, one experimental transect was performed each at the locations Avey2, Avey3, Avey4, and Avey6.

### 2.3 Post‐pollination pre‐zygotic isolation: fruiting success (RI_fruting)_

To measure post‐pollination pre‐zygotic barriers, manual intra‐/interspecific crosses were performed between the two species using 10 plants of *O. insectifera* and 7 plants of *O. aymoninii*. Intraspecific and interspecific crosses were performed with each of the two species (no plant was self‐pollinated). Post‐pollination pre‐zygotic isolation was quantified by counting the number of fruits (fresh fruits with seeds) on inter‐ and intraspecific crosses. RI_fruiting_ was calculated as 1 − (mean number of fruits in interspecific crosses/mean number of fruits in intraspecific crosses). In cases where interspecific crosses performed better than the intraspecific crosses (resulting in a negative value for RI), the reproductive isolation value was set to zero (Scopece et al., 2007). Finally, fruits were collected when they were ripe and dried in silica gel (Fluka).

### 2.4 Post‐zygotic isolation—embryo development (RI_embryo_)

To measure post‐zygotic isolation in the form of embryo development, seeds of the fruits were used for quantification of developed embryos. A random sample of 300 seeds from each fruit was examined under a light binocular microscope (Olympus SZH‐ILLD) at 64× magnification. Seeds with a well‐developed embryo and those without or with weakly developed embryos were counted. Well‐developed embryos were defined as visible black grains, within the transparent embryo sack. Weakly developed embryos were defined to be transparent like the embryo sack and much smaller than well‐developed ones. Post‐zygotic isolation due to absence of a developed embryo (RI_embryo_) was calculated as 1 − (mean number of developed embryos in interspecific crosses/mean number of developed embryos in intraspecific crosses, similar to Scopece et al. (2007). In cases where interspecific crosses performed better than the intraspecific crosses, the reproductive isolation value was set to zero (Scopece et al., 2007).

### 2.5 Molecular barcoding of mycorrhizal fungi

We collected root samples from 24 *O. insectifera* and 26 *O. aymoninii* individuals from five populations (Avey2‐6, on average 8 plants per population). Roots of the orchids were carefully excavated, and a ~1 cm long fragment of the root was removed. For each individual, roots were thoroughly washed, and on average 10 thin root sections (<0.2 μm in thickness) displaying mycorrhizal infection under a light microscope were obtained. DNA of the 396 resulting samples was extracted as in Schatz et al. (2010). Barcoding with the fungal ribosomal intergenic transcribed spacer (ITS) was performed using primers ITS1F and ITS4 (universal for fungi), ITS1 and ITS4Tul (specific for most tulasnelloids), as well as ITS1 and ITSTul2 (specific for some tulasnelloids, 5′‐TTCTTTTCCTCCGCTGAWTA‐3′), and thereafter sequenced as in Schatz et al. (2010). Operational taxonomic units (OTUs) were delineated at the 97% similarity threshold, and taxonomically affiliated using the BLAST algorithm (http://blast.ncbi.nlm.nih.gov/). To ascertain the phylogenetic position of Sebacinales OTUs, one longer sequence per each OTU was obtained with primers ITS3seb and TW13 as in Selosse, Dubois, and Alvarez (2009). One representative sequence per OTU was deposited in GenBank (GB accession numbers KF871201‐19).

### 2.6 Flower odor sampling and chemical analysis

Floral scent was collected from unpollinated and intact open flowers by cutting labella and extracting each in a 4‐ml glass vial (Supelco) filled with 0.5 ml dichloromethane (HPLC grade, Fluka) for 1 min while gently shaking. Subsequently, the labellum was removed and the samples stored at −28°C until analysis in a gas chromatograph. In total, scent extracts of 38 *O. aymoninii* and 48 *O. insectifera* plants were sampled during 2012 and 2013 from 5 populations (one flower per plant was used). For quantitative analysis of floral scent, a gas chromatograph (Agilent 6890N; Agilent Technologies, Santa Clara, CA, USA) with a flame ionization detector (FID) was used. One microlitre of each scent sample together with 1 μl of octadecane (10 ng/μl) as internal standard was injected splitless (closed split vent) at 50°C (1 min), followed by a programed increase in oven temperature to 300°C at a rate of 4°C/min. The GC was equipped with an Agilent 19091J‐431 column (15 m × 0.25 mm); hydrogen was used as carrier gas with a flow rate of 2.0 ml/min.

For identification of compounds, 60 scent samples (30 of each species) were additionally run on an Agilent GC with mass selective detection (Agilent 5975C; Agilent Technologies, Santa Clara, CA, USA). As above, 1 μl of the natural sample and 1 μl octadecane solution (1 ng/μl) as an internal standard were injected into the GC‐MS (gas chromatography‐mass spectrometry) system. For tentative identification of natural compounds, their mass spectra were compared with data reported in the NIST library and by Francke et al. (2000). For unequivocal structure assignments, mass spectra and gas chromatographic retention times of natural products were successfully compared with the following standards: tricosane, tetracosane, pentacosane, hexacosane, heptacosane, nonacosane (all purchased from Sigma‐Aldrich); (*Z*)‐9‐pentacosene, (*Z*)‐9‐heptacosane, (*Z*)‐9‐nonacosene (all from the stock collection of WF); octyl palmitate and nonyl palmitate (synthesized by WF through the reaction of palmitoyl chloride and the two primary alkohols following standard procedures). Additionally, four unknown compounds and docosenamide were included in the quantitative analysis due to their high abundance. Volatiles that were consistently detected in good signal‐to‐noise levels and all those that elicited EAD responses (in total 16 volatiles) were used for statistical comparison of relative amounts (amounts of individual components in relation to the total amounts of those 16 target compounds). Because heptacosane (C_27_) and nonyl palmitate were found to co‐elute on a DB‐5 column, all samples were run on a J&W 123‐7032 DB‐Wax (30 m × 0.25 μm) column with splitless injection at 50°C (1 min), followed by a programed increase in the oven temperature to 230°C at a rate of 10°C/min; hydrogen was used as carrier gas with a flow rate of 2.0 ml/min. The J&W 123‐7032 DB‐Wax column was used for heptacosane (C_27_) and nonyl palmitate to elute at different retention times, which were additionally identified and confirmed by running standards of both compounds. Based on the ratios of peak areas of the two compounds in these samples, the relative amount of each compound in all natural samples was estimated.

### 2.7 Electrophysiological recordings

Gas chromatographic analysis with electroantennographic detection (GC‐EAD; Schiestl & Marion‐Poll, 2002) of floral extracts was performed using a gas chromatograph (Agilent 6890 N, Agilent Technologies, Palo Alto, CA, USA) equipped with a heated outlet for electroantennographic recordings (effluent conditioning assembly, Syntech, Hilversum, the Netherlands). Antennal responses of *Andrena combinata* males were measured via EAD. No GC‐EAD experiments were performed with the pollinators of *O. insectifera* because none of them could be obtained in the field. For EAD recordings*,* the tip of the excised antenna was abscised and the antenna was mounted between two glass capillaries filled with insect Ringer solution mounted on a micro‐manipulator (MP‐12, Syntech). The electrode at the base of the antenna was grounded via an Ag/AgCl wire and the electrode at the distal end of the antenna was connected via a signal interface box (Syntech) to a personal computer. Up to 5 μl of *O. aymoninii* flower extract was injected splitless at 50°C (1 min) into the GC followed by heating to 300°C with a rate of 10°C/min. The GC was equipped with an HP‐5 column (0.32 mm diameter, 0.25 μm film thickness, 30 m length) and a flame ionization detector (FID). Hydrogen was used as carrier gas. A GC effluent splitter (Agilent G2855 Deans Switching System, Agilent Technologies, Palo Alto, CA, USA) was used to direct 50% of the eluate, which was admixed to a purified and humidified air stream, over the excised antenna. EAD signals and FID responses were simultaneously recorded using Syntech software. EAD responses were judged “real” if reproducible in at least four bee individuals. Compounds releasing EAD responses were identified by comparison of retention times of samples with those of synthetic standard compounds.

### 2.8 Behavioral assays

Behavioral assays were conducted to test whether the production of key volatiles can induce a pollinator switch. For all assays, freshly picked plants with unpollinated flowers were used; manipulations were done for all flowers of an inflorescence. Flowers of *O. insectifera* were manipulated by drippling 10 μl of a scent mixture in hexane (25 ng/μl (*Z*)‐9‐C25, 27 ng/μl, (*Z*)‐9‐C27, 7 ng/μl nonyl palmitate, and 5 ng/μl octyl palmitate) onto the flower labellum using a glass syringe. These four compounds were chosen as they were the EAD‐active compounds in *O. aymoninii* and were primarily produced in this species. The amounts of the compounds were chosen to mimic those found in natural flower extracts of *O. aymoninii,* as measured in scent extracts analyzed by GC‐FID. As a negative control, unpollinated *O. insectifera* flowers treated with 10 μl pure hexane were used. Non‐manipulated flowers of *O. aymoninii* served as positive controls for the assays. The plants were placed in 15‐mL falcon tubes filled with water along the patrol pathways of male *Andrena combinata* bees (bushes, pine trees) randomly in a distance of 0.2 m from each other. Each experimental set up consisted of two plants from every treatment with an equal number of open flowers. The number of approaches (male bees that approached the flower to a distance of ca 10 cm or closer without landing) as well as landings (including attempted copulations) were recorded visually. Each group of plants was assayed for 30 min, then plants were changed and the test location moved for a few meters. These experiments were performed between 11:00 and 15:00 hrs at the population Avey2 (on 4 days) and Avey6 (on 1 day) during May and June 2013. A reciprocal experiment with the digger wasp‐pollinator of *O. insectifera* could not be done, as those pollinators were never observed in the field.

### 2.9 Fruit set and density

In 2013, six populations (avey2, 3, 4, 5, 6, and 8) were surveyed for fruit set. A total of 157 *O. aymoninii* and 143 *O. insectifera* plants were chosen randomly at the beginning of the flowering season, marked, and measured for plant height and number of conspecific plants within a 2 m radius. At the end of the flowering season, approximately 1 month later, the total number of flowers and fruits of the marked plants were counted. Because of the typically low fruit set in sexually deceptive orchids, plants are usually limited by pollinators rather than resources in their fruit set, and fruit set is a good proxy for pollination success (Schiestl, unpublished data; Scopece, Cozzolino, Johnson, & Schiestl, 2010).

### 2.10 Phylogenetic analysis

To uncover the phylogenetic relationships within the *O. insectifera* group, we Sanger‐sequenced three nuclear markers (*BGP*,* LACS*, and *LFY* selected as the most variable regions from the original dataset of Breitkopf et al. (2015) for 18 *Ophrys* specimens belonging to the *O. insectifera* lineage (namely 3 *O. aymoninii*, 3 *O. subinsectifera*, and 12 *O. insectifera*) plus one accession of *O. garganica* used as outgroup. The combined alignment was analyzed using Bayesian inference (MrBayes v.3.1.2), with the un‐partitioned dataset and by employing the GTR+Γ model of molecular evolution according to Breitkopf et al. (2015). Bayesian analysis was conducted with a single runs of a Markov‐chain Monte Carlo (MCMC) chain for one million generations with tree sampling every 500 generations (CBSU BioHPC, Cornell University). Runs converged at split frequencies below 0.01 after 600,000 generations. The combined dataset had a length of 957 base pairs and Bayesian inference produced a single tree.

### 2.11 Ploidy level

To exclude differences in ploidy as a reproductive barrier, the relative ploidy level of pollinia from both species was measured. In 2010, both pollinia were sampled of one flower from 12 *O. insectifera* plants and 11 *O. aymoninii* plants in S‐France at the Avey‐3 population. Relative ploidy level was analyzed using flow cytometry following Xu et al. (2011). Two pollinia were chopped and mashed together with approximately 25‐mm^2^ leaf material of *Phaseolus coccineus* (2*n*, 1C = 1.01 ± 0.4 pg) which served as internal standard (IS), with a sharp razor blade in 1‐mL ice‐cold Baranyi's solution (0.1 mol/L citric acid, 0.5% Triton X‐100). After filtering the suspension through a 30 μm CellTrics^*®*^ disposable filter (Partec GmbH, Münster, Germany), the filtrate was centrifuged (5 min, 380 × *g*, room temperature) using a Sorvall^*®*^ RMC 14 centrifuge (Kendro Revco Lindberg Heraeus Sorvall, Asheville, NC). After removal of supernatant, nuclei were resuspended in 40 μl of ice‐cold Baranyi's solution. In total, 160 micro liters of Otto II solution (0.4 mol/L Na2HPO4) supplemented with DAPI (4′, 6‐diamidino‐2‐phenylindole; final concentration: 4 μg/ml) were added and relative fluorescence intensity was recorded using a Cell Lab QuantaTM SC‐MPL flow cytometer (Beckman Coulter, Fullerton, Canada) with a mercury arc lamp. Only samples with pollinia peaks of at least 1,000 counts and a coefficient of variation of <10% were analyzed. To determine relative ploidy level of the two species, the ratios between the median of pollinia peaks and the median of IS peaks were calculated.

### 2.12 Statistical analysis

#### 2.12.1 Fecundity and density

Mean fruit set per population was calculated as the average number of fruits produced by all surveyed plants in each population. Relative fruit set was calculated by dividing the number of fruits produced by each individual by the mean fruit set of its population. Fruits per plant was calculated by dividing the number of fruits of each individual by its total number of flowers. To compare the measured parameters (Table 1) between the species, a general linear model was run with each plant parameter as dependent, species as fixed, and population as random factor. To analyze the impacts of all measured parameters on fecundity, a general linear model was calculated with relative fruit set as a dependent variable, species as fixed, population as random factor, and “number of conspecifics,” “plant height,” and “total number of flowers” as covariates. The interaction between “no. of conspecifics” and species was also included, to assess whether density‐dependent fruit set differs between species. All covariates were z‐transformed (mean = 0, *SD* = 1) on species and population level before analysis. Because neither species nor population had a significant effect of relative fruit set, we also calculated a multiple linear regression with relative fruit set as dependent, and “no. of conspecifics,” “plant height,” and “no. of flowers” as explanatory variables.

**Table 1:** Mean (±*SD*) values of traits measured in the two species in six natural populations. None of the traits was consistently different between the species, but several (maked with an asterisk) showed a significant interaction between species and population (GLM, *p* ≤ .001)

	*Ophrys insectifera*	*Ophrys aymoninii*
No. of flowers	5.09 ± 1.70	4.57 ± 1.69
Plant height (cm)	32.08 ± 7.48	20.50 ± 5.39*
No. of fruits	0.61 ± 1.22	1.57 ± 1.82*
Fruits per flowers	0.12 ± 0.19	0.33 ± 0.35*
No. of conspecifics within 2 m radius	4.83 ± 4.11	5.67 ± 7.56*

#### 2.12.2 Floral scent

Differences in the relative amounts of individual floral scent compounds between the two species were analyzed using Mann–Whitney *U*‐tests. In addition, we transformed our matrix of relative amounts of compounds (originally in % of the total blend) with a Hellinger transformation, which is a relativization by row (sample unit) totals, followed by taking the square root of each element in the matrix, to make the floral scent data that contained many zero values (e.g., compounds absent in certain individuals, but present in others) suitable for multivariate analysis (Legendre & Gallagher, 2001; Legendre & Legendre, 1998). We then performed an analysis of similarities (ANOSIM) using the average Bray–Curtis distances among samples of the Hellinger‐transformed matrix and 1,000 permutations with the *vegan* package (version 2.0–5; (Oksanen et al., 2012)) in R to statistically test if the two *Ophrys* species differed in floral scent. To characterize and visualize the floral scent dissimilarities between species, we performed a non‐metric multi‐dimensional scaling (nMDS) ordination based on a matrix of Bray–Curtis dissimilarities still based on the relative proportions of odor compounds. The appropriateness of the nMDS results was determined by comparing, in a Shepard diagram, where the distances among samples in the ordination plot with the original distances, and the stress value generated with the nMDS analysis reflects how well the ordination summarizes the observed distances among the samples. The nMDS analysis was performed with the *vegan* package (version 2.0–5; (Oksanen et al., 2012)) in R.

#### 2.12.3 Behavioral assays, mycorrhiza

The numbers of behavioral responses of pollinators toward scent manipulated *O. insectifera*, negative and positive controls were analyzed through Chi^2^ tests with Bonferroni correction (α = 0.017, equal frequencies expected, for both approaches and landing). Differences in the occurrence of mycorrhiza fungi between *O. insectifera* and *O. aymoninii* were analyzed with generalized linear model with binomial distribution; mycorrhiza presence/absence was used as dependent variable and species as explanatory variable. Due to absence or extremely low abundance of mycorrhizal fungi for T3 and S2, no statistical analysis could be performed for those strains.

## 3 RESULTS

### 3.1 Pre‐zygotic isolation (floral isolation)

**Figure 2:**
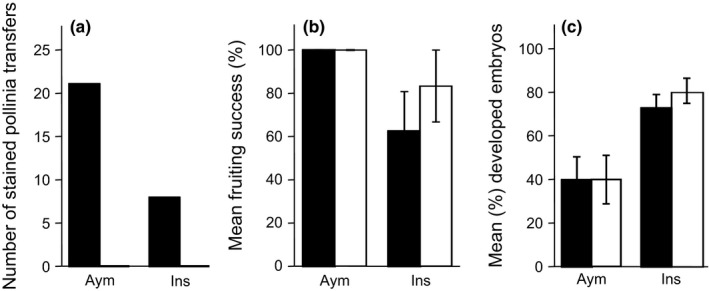
Effectiveness of different reproductive barriers in *Ophrys aymoninii* (Aym) and *O. insectifera* (Ins)*;* black bars: intraspecific pollinations/crosses, white bars: interspecific pollinations/crosses. (a) Floral isolation, pollinia transfers; significantly more intra‐ than interspecific transfers were found (binomial test, Aym: *p* < .001; Ins: *p* = .008). (b) Pre‐zygotic–post‐pollination isolation, fruit set; (c) Post‐zygotic isolation, developed embryos. Error bars correspond to standard error of mean; for both b and c, no differences were found between inter‐ and intraspecies crosses

The phenology of *O. aymoninii* and *O. insectifera* flowers was found to broadly overlap, as both species were in full bloom during all seasons of field work (Daniel Gervasi personal observations). In the 3 years where floral isolation plots were set up, we detected only few pollen transfers, which is in accordance with the low pollination success typically found in sexual mimics. A total of 29 flowers (8 *O. insectifera* and 21 *O. aymoninii*) received stained massulae, and all these transfers were between plants of the same species; not a single between‐species transfer was observed (Figure 2a). Thus, both species had an estimated RI_floral_ value of 1 indicating complete or at least very strong reproductive isolation.

### 3.2 Post‐pollination pre‐zygotic isolation (fruiting success)

From 26 hand crosses, the six intra‐ and six interspecific crosses with *O. aymoninii* as pollen receiver led to equal fruit set (Figure 2b), resulting in a RI_fruiting_ value of 0. In *O. insectifera*, the six interspecific crosses had an even higher fruiting success than the eight intraspecific crosses resulting in a negative RI_fruiting_ value of −0.333, subsequently set to zero. Thus, no reproductive barrier at this stage was found (Figure 2b).

### 3.3 Post‐zygotic isolation (embryo development)

In both species, interspecific crosses showed a tendency to higher yield of seeds with well‐developed embryos than the intraspecific crosses, albeit not significant (Figure 2c). For both species, a negative RI_embryo_ value was obtained (*O. ins* = −0.111, *O. aym* = −0.006) and, therefore, set to zero, indicating no reproductive isolation through reduced embryo development. The two species also showed to have the same overall ploidy level (Fig. S2) increasing the possibility of hybrids to be fertile.

### 3.4 Mycorrhizal fungi

**Figure 3:**
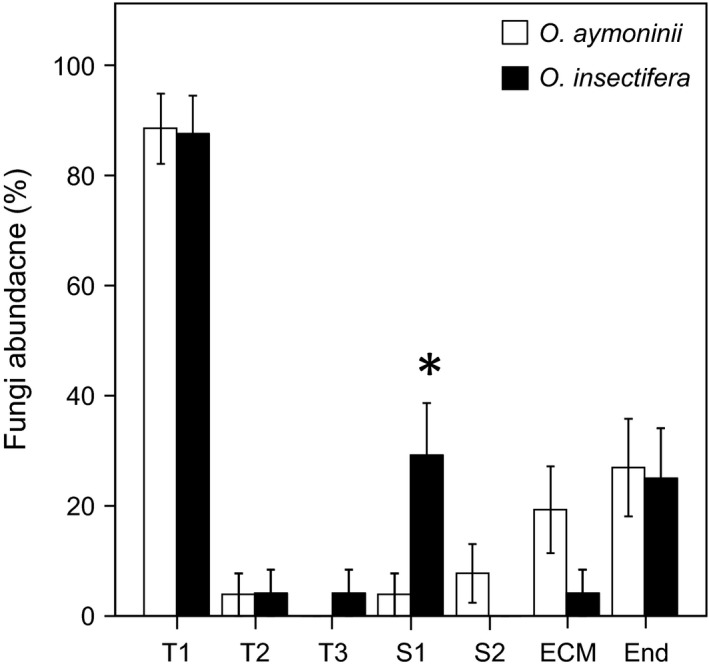
Relative abundance (±standard err) of specific fungi in *Ophrys aymoninii* (*N* = 26) and *O. insectifera* (*N* = 24) plants. T1‐3 = *Tulasnella*, S1‐2 =  Sebacinales, ECM = ectomycorrhizal fungi, End = endophytic fungi. Asterisk indicates significant differences in relative fungi abundance between the two species. No statistical tests were performed for T3 S2 because of their low abundance

Barcoding identified the Tulasnellaceae operational taxonomic unit (OTU) T1 (GB accession number KF871201) as the most frequent mycorrhizal fungus in both orchid species, detected in 23 (out of 24) and 21 (out of 26) individuals of *O. insectifera* and *O. aymoninii*, respectively (Figure 3, Table S1). Other rhizoctonias included two Tulasnellaceae OTUs (T2 and T3; KF871202‐3) and two Serendipitaceae OTUs (S1 and S2; KF871204‐5; Table S2). All rhizoctonias OTUs were found on both host orchids, with exception of T3 (on one *O. insectifera* individual only) and S2 (on two *O. insectifera* individuals only; Figure 3). Barcoding also revealed OTUs of endophytic fungi (KF871206‐14) or common mycorrhizal fungi of forest trees (ectomycorrhizal fungi, such as *Tricholoma*,* Rhizopogon*, and *Russula*; KF871215‐19; Table S1), unlikely to be truly orchid mycorrhizal fungi (Dearnaley et al., 2013). GLM analysis revealed no difference in frequency of individuals with T1 (*df* = 1, Wald *X* ^2^ = 0.011; *p *=* *.917), T2 (*df* = 1, Wald *X* ^2^ = 0.003; *p *=* *.954), endophytic fungi (*df* = 1, Wald *X* ^2^ = 2.971; *p *=* *.085), and ectomycorrhizal fungi (*df* = 1, Wald *X* ^2^ = 0.024; *p *=* *.877). The only significant difference was found for Serendipitaceae S1 (Figure 3; *df* = 1, Wald *X* ^2^ = 6.392; *p *=* *.011). Based on this large overlap, differences of mycorrhizal partners are unlikely to form a reproductive barrier.

### 3.5 Fruit set and density

None of the measured traits (plant height, fruits, and density) differed consistently between the species, but for several of them, a highly significant interaction between species and population was found (Table 1). In our general linear model, “number of conspecifics” (=density) was the only factor with a significant effect on relative fruit set (Table 2). In a multivariate regression, it was shown that the effect of density on relative fruit set was significantly negative (Table 3).

**Table 2:** Effects of different factors on relative fruit set of the two *Ophrys* species analyzed by a general linear model. Only the number of conspecifics (within a radius of 2 m), used as a measure of density, had a significant effect on relative fruit set in the two species. Statistically significant values are given in bold

Source	*df*	*F*	*p*
Species	1, 289	0.059	.808
Population	5, 289	0.119	.988
**No. of conspecifics**	**1, 289**	**5.350**	**.021**
No. of flowers	1, 289	3.331	.069
Plant height	1, 289	0.113	.717
Species × conspecifics	1, 289	1.523	.218

**Table 3:** Multiple linear regression with relative fruit set as dependent variable, and number of consepcifics, number of flowers, and plant height as explanatory variables. Number of conspecifics, used as a proxy for density, had a significant negative effect on relative fruit set. Statistically significant values are given in bold

Traits	β (±*SE*)	*p*
**No. of conspecifics**	**−0.228 ± 0.1**	**.024**
No. of flowers	0.218 ± 0.12	.070
Plant height	0.045 ± 0.12	.709

### 3.6 Floral scent and GC‐EAD

**Figure 4:**
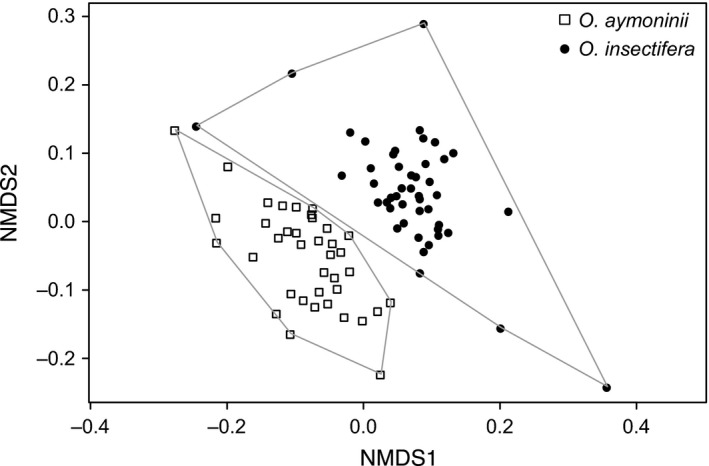
Differences in relative amounts of floral volatiles between the two *Ophrys* species shown by an nMDS biplot of a Bray–Curtis dissimilarity matrix (stress value = 0.14). In this analysis, no overlap between the sent bouquets of the two species was found

Of all floral volatiles in the samples, the wax esters, octyl palmitate and nonyl palmitate, as well as the alkenes, (*Z*)‐9‐pentacosene and (*Z*)‐9‐heptacosene, were found to elicit EAD responses in *Andrena combinata* males (Fig. S3); one additional compound, tricosane, also elicited reproducible EAD responses, but this compound was found in both species in the same amounts (Table 4) and was thus not considered for the bioassays. Overall, of the 16 most abundant floral volatiles, 12 were chemically identified (Table 4). Of these 16 compounds, 13 were found to differ significantly between species (Table 4). The overall bouquet was different, too (Figure 4). The most striking differences were found within the relative amounts of EAD‐active esters and alkenes. Octyl palmitate and nonyl palmitate, as well as (*Z*)‐9‐pentacosene and (*Z*)‐9‐heptacosene, were found to be present in significantly higher amounts in *O. aymoninii* than *O. insectifera* (Table 4).

**Table 4:** Mean relative amounts (% ± *SE*) of 16 volatiles in *O. aymoninii* (*N* = 38) and *O. insectifera* (*N* = 46) arranged after their retention time (shortest–longest). Compounds in bold are electrophysiological active volatiles based on GC‐EAD with male *Andrena combinata* bees and *O. aymoninii* scent extracts. Different superscripts indicate significant differences between the species (Mann–Whitney *U*‐Test, *p* < .05)

Compounds	*O. aymoninii*	*O. insectifera*
Unknown 1	2.282 ± 0.172^a^	3.644 ± 0.273^b^
**Tricosane**	19.998 ± 0.657^a^	19.929 ± 0.820^a^
Tetracosane	2.925 ± 0.172^a^	2.655 ± 0.079^b^
**(** ***Z*** **)‐9‐Pentacosene**	7.415 ± 0.561^a^	1.076 ± 0.067^b^
Pentacosane	12.104 ± 0.300^a^	13.683 ± 0.369^b^
**Octyl palmitate**	0.675 ± 0.063^a^	0.066 ± 0.028^b^
Hexacosane	0.674 ± 0.033^a^	0.994 ± 0.042^b^
Unknown 2	1.183 ± 0.046^a^	1.160 ± 0.041^a^
**(** ***Z*** **)‐9‐Heptacosene**	19.448 ± 0.693^a^	14.967 ± 0.478^b^
**Nonyl palmitate**	2.217 ± 0.152^a^	0.429 ± 0.091^b^
Unknown 3	2.635 ± 0.155^a^	3.776 ± 0.184^b^
Heptacosane	4.524 ± 0.121^a^	7.119 ± 0.279^b^
Docosenamid	5.074 ± 1.168^a^	5.173 ± 1.105^a^
Unknown 4	9.340 ± 0.595^a^	12.795 ± 0.742^b^
(*Z*)‐9‐Nonacosene	8.397 ± 0.466^a^	10.738 ± 0.429^b^
Nonacosane	1.112 ± 0.052^a^	1.797 ± 0.118^b^

### 3.7 Behavioral assays

**Figure 5:**
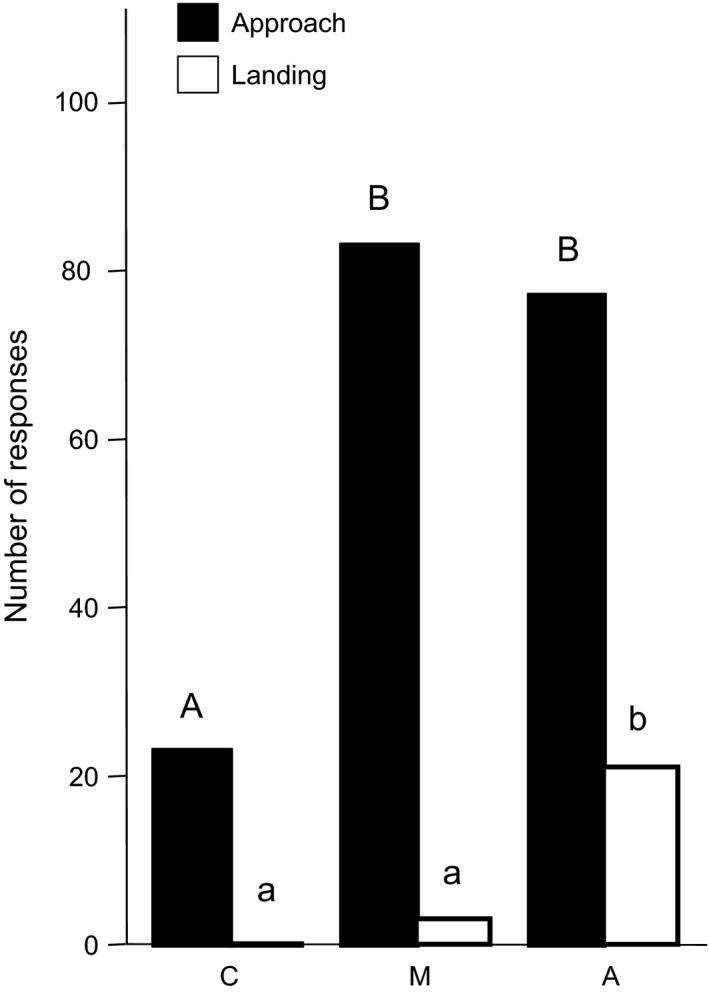
Behavioral assays with scent‐manipulated *Ophrys insectifera* plants and male *Andrena combinata* bees in the field. Assayed plants: C: negative control (*O. insectifera* with solvent only), M: *O. insectifera* flowers manipulated with four EAD‐active *O. aymoninii* volatiles ((*Z*)‐9‐C25, (*Z*)‐9‐C27, nonyl palmitate, octyl palmitate), A: positive control, un‐manipulated *O. aymoninii* flowers. Different letters above the bars indicate significant differences among the treatments (Chi^2^ test, *p* ≤ .017)

Our behavioral assays showed that the blend of four GC‐EAD active scent components (octyl palmitate, nonyl palmitate, (*Z*)‐9‐pentacosene, and (*Z*)‐9‐heptacosene), produced primarily in *O. aymoninii,* is crucial for the attraction of *Andrena combinata* males. Our application of these compounds onto flowers of *O. insectifera* (“scent manipulation”) significantly increased approaches by *A. combinata* males (Figure 5, Χ^2^_1_ = 33.962; *p* < .001) compared to negative controls (*O. insectifera* flowers with solvent only). The scent‐manipulated *O. insectifera* flowers even had the same number of approaches than positive controls (non‐manipulated *O. aymoninii* flowers; Figure 5, Χ^2^_1_ = 0.225; *p* = .635). Scent‐manipulated *O. insectifera* flowers received three landings, one of them leading to a copulation attempt (Figure 1e). Despite the fact that three landings were not statistically different from the zero landings on negative controls, and significantly less than the 21 landings (including copulation‐attempts) observed on positive controls (Figure 5), this result shows that the four compounds can induce the attraction of a new pollinator and the behavior (copulation attempt) necessary for uptake or deposition of pollinia.

### 3.8 Phylogenetic analysis

Our phylogenetic analysis using three nuclear markers (*BGP*,* LACS*, and *LFY*) from 18 specimens of the *O. insectifera* group showed no clear species clustering between the species. This was as expected from the morphology‐based taxonomic classification and suggests a close relationship between the three members of the *O. insectifera* group (Fig. S4). In contrast, a moderate geographical clustering was evident in the analyses. Results found here mirror those already found by Breitkopf et al. (2015) in all terminal clades of their *Ophrys* phylogenetic analysis and point toward an incomplete lineage sorting scenario as consequence of very recent radiation of species groups in this genus.

## 4 DISCUSSION

Experimental investigations on the evolution and nature of reproductive isolation barriers can provide insights into the process of diversification (Coyne & Orr, 1998, 2004; Moyle et al., 2004; Schemske, 2010; Scopece et al., 2007, 2008; Widmer et al., 2009). In our study, floral isolation mediated by floral scent appears to be the only significant barrier to gene flow between two recently diverged orchid species. Although the predominant importance of ethological floral isolation in sexual mimics has also been shown in other species (Scopece et al., 2007; Sedeek et al., 2014; Whitehead & Peakall, 2014; Xu et al., 2011), our study adds information on the traits underlying floral isolation and shows a negative association between fruit set and plant density, a situation that may favor a pollinator switch. In addition, it considers mycorrhizal fungi as a factor for species isolation, which has rarely been done in orchids and never before in the genus *Ophrys*.

Species‐specific mycorrhizal fungi may mediate isolation in two ways: first, as a post‐zygotic barrier, hybrids may suffer low fungal recruitment success and hence low germination or seedling survival (Jacquemyn et al., 2011); second, non‐randomly distributed fungal species may also influence the habitat preference of their host species, leading to ecological segregation. Our investigations detected a broad sharing of mycorrhizal fungi, with a marked preference for one Tulasnellaceae species. Using species delineation based on 3% ITS divergence is unlikely to have masked cryptic Tulasnellaceae species: first, this is a usual threshold and ITS species delineation is validated in Tulasnellaceae by the fact that it is congruent with other genes (Linde, Phillips, Crisp, & Peakall, 2014); second, lowering the threshold to 1.5% did not change OTU delineation in our work. This fungus family is common in several *Ophrys* species (Jacquemyn, Brys, Waud, Busschaert, & Lievens, 2015; Pecoraro, Girlanda, Liu, Huang, & Perotto, 2015). Only a small difference in mycorrhizal partners was found earlier in closely related species of the genus *Orchis* in sympatry (Jacquemyn et al., 2011), and sexually deceptive orchids of the genus *Chiloglottis* were shown to share a narrow taxonomic group of *Tulasnella* fungi (Roche et al., 2010). Furthermore, a recent study in the sexually deceptive orchid *Caladenia* was also showing a strong overlap in mycorrhizal partners suggesting little contribution to reproductive isolation (Phillips, Barrett, Dalziell, Dixon, & Swarts, 2016). The consequence of the sharing of mycorrhizal fungi makes specificity of mycorrhizal symbiosis unlikely to contribute to reproductive isolation, or to enhance pre‐zygotic barriers.

Floral isolation, thus, remains the hallmark of species separation in sexual mimics. Whereas in many plant systems with prominent floral isolation, morphologic and ethological components act synergistically (Dell'Olivo, Hoballah, Gubitz, & Kuhlemeier, 2011; Grant, 1994; Kay, 2006; Sun et al., 2015; Tang, Yu, Sun, & Huang, 2007), sexual mimics usually rely on specific pollinator attraction alone (Xu et al., 2011; Peakall & Whitehead, 2014; Sedeek et al., 2014; Whitehead & Peakall, 2014; but see (Gögler et al., 2009; Gaskett, 2012; de Jaeger & Peakall, 2015). Such species specificity in pollinator attraction is usually driven by differences floral scent chemistry (Johnson & Schiestl, 2016; Okamoto, Okuyama, Goto, Tokoro, & Kato, 2015). In *Ophrys‐*species pollinated by *Andrena* bees, differences in the relative proportions of alkanes and especially alkenes are considered decisive for attraction of different pollinator species (Ayasse et al., 2011; Schiestl et al., 2000; Xu, Schlüter, & Schiestl, 2012), but wax esters have also been found important for pollinator behavior (Ayasse et al., 2000). Our finding of higher proportions of two alkenes and wax esters in *O. aymoninii* is in agreement with early investigations (Borg‐Karlson, Groth, Ågren, & Kullenberg, 1993) that also detected higher amounts of esters in *O. aymoninii* plants compared to *O. insectifera*. We show here that two esters and alkenes are sufficient to increase the attractiveness toward another pollinator significantly; this suggests that whereas *Ophrys* species typically differ in a range of scent components, key differences for specific pollinator attraction may be less complex, even reminiscent of the chemical simplicity in Australian genus *Chiloglottis*, where differences in single compounds are sufficient to trigger pollinator switches (Peakall et al., 2010).

The classical Grant–Stebbins model of pollinator‐driven speciation assumes pollinators are heterogeneously distributed and thus plants growing in different areas are under selection to switch pollinator (Grant, 1994; Grant & Grant, 1965; Van der Niet, Pirie, et al., 2014). Alternatively, adaptation to new pollinators may be fueled by competition for pollination in large plant populations, in the form of negative density‐dependent fecundity (Waser & Campbell, 2004). In our study, relative fruit set was indeed negatively associated with number of conspecifics growing close by, suggesting plants have better fruit set when growing isolated or being rare, given similar pollinator abundances. Lower fruit set in dense patches can be explained by negative associative learning of pollinators that unsuccessfully attempted to copulate with flowers (Ayasse et al., 2000; Peakall, 1990; Wong & Schiestl, 2002). The avoidance of patches of plants may lead to less visits to each individual plant in a dense population compared to sparsely distributed individuals. Such competition for pollination may promote a pollinator switch because individuals attracting a new pollinator are necessarily rare in the beginning of this process, and thus may enjoy increased pollination success.

Our data suggest that the requirement for the attraction of a novel *Andrena‐*pollinator in *O. insectifera* is a mutation leading to elevated alkene/ester production. As yet we do not know, however, whether elevated alkenes/esters also reduce the attraction of the pollinator of *O. insectifera*, which is a necessary prerequisite for isolation against backcrossing into wild‐type *O. insectifera*. In previous experiments in sexual mimics of the genus *Ophrys* and *Chiloglottis*, however, it has been shown that hetero‐specific scent clearly reduces pollinator attraction (Peakall et al., 2010; Xu, Schlüter, & Schiestl, 2012). Nevertheless, some overlap in pollinators is likely during the switching phase, unless antagonistic pleiotropy between attractive scent compounds would prevent a phenotype emitting a blend of both. This major obstacle to a sympatric speciation scenario, namely recombination through gene flow breaking down associations between co‐adapted alleles (Coyne & Orr, 2004), would be prevented by a mono‐ or oligogenetic basis of the trait mediating floral isolation, in our case the alkene/ester production.

## 5 CONCLUSION

This study shows that floral isolation, that is, specific attraction of pollinators through floral odor, apparently acts as the sole reproductive barrier for maintaining species integrity in the *O. insectifera* group. Moreover, this study indicates that female reproductive success was negatively associated with density and that few scent compounds can induce—at least occasional—copulation attempts by a new pollinator. However, for a better understanding of speciation scenarios in sexual mimics, a better resolved phylogenetic framework is desirable, to confidently assign recently diverged pairs of sister species within the flock of genetically often very similar species (Breitkopf et al., 2015). Furthermore, in our specific study system, a better understanding of the chemical ecology of the pollinator of *O. insectifera* is needed to predict its behavioral responses to variation in floral scent. Such data would allow to more confidently predict patterns of introgression during the establishment of distinct scent types. Finally, a better understanding of the molecular background of key traits for floral isolation (Sedeek et al., 2016) will help unravel origin and maintenance of floral scent differences even in the face of occasional gene flow, and thus better understand speciation in this intriguing group of plants.

## CONFLICT OF INTEREST

None declared.

## Supporting information


** **

** **

** **

** **

** **

## References

[ece33147-bib-0001] Ayasse, M. , Schiestl, F. P. , Paulus, H. F. , Löfstedt, C. , Hansson, B. , Ibarra, F. , & Francke, W. (2000). Evolution of reproductive strategies in the sexually deceptive orchid *Ophrys sphegodes*: How does flower‐specific variation of odor signals influence reproductive success? Evolution, 54, 1995–2006. 11209776 10.1111/j.0014-3820.2000.tb01243.x

[ece33147-bib-0002] Ayasse, M. , Stökl, J. , & Francke, W. (2011). Chemical ecology and pollinator‐driven speciation in sexually deceptive orchids. Phytochemistry, 72, 1667–1677. 21497864 10.1016/j.phytochem.2011.03.023

[ece33147-bib-0003] Bateman, R. M. , Rudall, P. J. , Bidartondo, M. I. , Cozzolino, S. , Tranchida‐Lombardo, V. , Carine, M. A. , & Moura, M. (2014). Speciation via floral heterochrony and presumed mycorrhizal host switching of endemic butterfly orchids on the Azorean archipelago. American Journal of Botany, 101, 979–1001. 24907253 10.3732/ajb.1300430

[ece33147-bib-0004] Borg‐Karlson, A. K. , Groth, I. , Ågren, L. , & Kullenberg, B. (1993). Form‐specific fragrances from *Ophrys insectifera* L. (Orchidaceae) attract species of different pollinator genera. Evidence of sympatric speciation? Chemoecology, 4(1), 39–45.

[ece33147-bib-0005] Breitkopf, H. , Onstein, R. E. , Cafasso, D. , Schlüter, P. M. , & Cozzolino, S. (2015). Multiple shifts to different pollinators fuelled rapid diversification in sexually deceptive *Ophrys* orchids. New Phytologist, 207, 377–389. 25521237 10.1111/nph.13219

[ece33147-bib-0006] Cortis, P. , Vereecken, N. J. , Schiestl, F. P. , Lumaga, M. R. B. , Scrugli, A. , & Cozzolino, S. (2009). Pollinator convergence and the nature of species’ boundaries in sympatric Sardinian *Ophrys* (Orchidaceae). Annals of Botany, 104, 497–506. 19001428 10.1093/aob/mcn219 PMC2720645

[ece33147-bib-0007] Coyne, J. A. , & Orr, H. A. (1989). Patterns of speciation in *Drosophila* . Evolution, 43, 362–381. 28568554 10.1111/j.1558-5646.1989.tb04233.x

[ece33147-bib-0008] Coyne, J. A. , & Orr, H. A. (1998). The evolutionary genetics of speciation. Philosophical Transactions of the Royal Society of London Series B‐Biological Sciences, 353, 287–305. 10.1098/rstb.1998.0210 PMC1692208 9533126

[ece33147-bib-0009] Coyne, J. A. , & Orr, H. A. (2004). Speciation. Sunderland, MA: Sinauer.

[ece33147-bib-0010] Dearnaley, J. W. D. , Martos, F. , & Selosse, M.‐A. (2013). Orchid mycorrhizas: Molecular ecology, physiology, evolution and conservation aspects In Hock B. (Ed.), The Mycota IX: Fungal associations (pp. 207–230). Berlin Heidelberg: Springer.

[ece33147-bib-0011] Dearnaley, J. W. D. , Perotto, S. , & Selosse, M.‐A. (2016). Structure and development of orchid mycorrhizas In Martin F. (Ed.), Molecular mycorrhizal symbiosis (pp. 63–68). Berlin Heidelberg: Springer.

[ece33147-bib-0012] Delforge, P. (2005). Orchids of Europe, North Africa and the Middle East. Paris: Delachaux and Niestlé.

[ece33147-bib-0013] Dell'Olivo, A. , Hoballah, M. E. , Gubitz, T. , & Kuhlemeier, C. (2011). Isolation barriers between *Petunia axillaris* and *Petunia integrifolia* (Solanaceae). Evolution, 65, 1979–1991. 21729053 10.1111/j.1558-5646.2011.01279.x

[ece33147-bib-0014] Devey, D. S. , Bateman, R. M. , Fay, M. F. , & Hawkins, J. A. (2008). Friends or relatives? Phylogenetics and species delimitation in the controversial European orchid genus *Ophrys* . Annals of Botany, 101, 385–402. 18184645 10.1093/aob/mcm299 PMC2701817

[ece33147-bib-0115] Francke, W.. , Lubke, G. , Schroder, W. , Reckziegel, A. , Imperatriz‐Fonseca, V. , Kleinert, A. , ... Engels, W. (2000). Identification of oxygen containing volatiles in cephalic secretions of workers of Brazilian stingless bees. J. Braz. Chem. Soc, 11, 562–571.

[ece33147-bib-0015] Fritz, A. L. , & Nilsson, L. A. (1994). How pollinator‐mediated mating varies with population‐size in plants. Oecologia, 100, 451–462. 28306934 10.1007/BF00317867

[ece33147-bib-0016] Gaskett, A. C. (2011). Orchid pollination by sexual deception: Pollinator perspectives. Biological Reviews, 86, 33–75. 20377574 10.1111/j.1469-185X.2010.00134.x

[ece33147-bib-0017] Gaskett, A. C. (2012). Floral shape mimicry and variation in sexually deceptive orchids with a shared pollinator. Biological Journal of the Linnean Society, 106, 469–481.

[ece33147-bib-0018] Gervasi, D. , & Schiestl, F. P. (2017). Real time divergent evolution in plants driven by pollinators. Nature Communications, https://doi.org/10.1038/ncomms14691. 10.1038/ncomms14691 PMC5424062 28291771

[ece33147-bib-0019] Gögler, J. , Stökl, J. , Sramkova, A. , Twele, R. , Francke, W. , Cozzolino, S. , Cortis, P. , Scrugli, A. , & Ayasse, M. (2009). Menage a trois‐two endemic species of deceptive orchids and one pollinator species. Evolution, 63, 2222–2234. 19473395 10.1111/j.1558-5646.2009.00712.x

[ece33147-bib-0020] Grant, V. (1994). Modes and origins of mechanical and ethological isolation in angiosperms. Proceedings of the National Academy of Sciences of the United States of America, 91, 3–10. 11607448 10.1073/pnas.91.1.3 PMC42875

[ece33147-bib-0021] Grant, V. , & Grant, K. A. (1965). Flower pollination in the Phlox family. New York, NY: Columbia University Press.

[ece33147-bib-0022] Jacquemyn, H. , Brys, R. , Cammue, B. P. A. , Honnay, O. , & Lievens, B. (2011). Mycorrhizal associations and reproductive isolation in three closely related *Orchis* species. Annals of Botany, 107, 347–356. 21186239 10.1093/aob/mcq248 PMC3043927

[ece33147-bib-0023] Jacquemyn, H. , Brys, R. , Waud, M. , Busschaert, P. , & Lievens, B. (2015). Mycorrhizal networks and coexistence in species‐rich orchid communities. New Phytologist, 206, 1127–1134. 25614926 10.1111/nph.13281

[ece33147-bib-0024] de Jaeger, M. L. , & Peakall, R. (2015). Does morphology matter? An explicit assessment of floral morphology in sexual deception. Functional Ecology, 30, 537–546. https://doi.org/10.1111/1365-2435.12517.

[ece33147-bib-0025] Johnson, S. D. (2006). Pollinator driven speciation in plants In Harder L. D., & Barrett S. C. H. (Eds.), Ecology and evolution of flowers (pp. 295–310). Oxford: Oxford University Press.

[ece33147-bib-0026] Johnson, S. D. , & Schiestl, F. P. (2016). Floral mimicry. Oxford: Oxford University Press.

[ece33147-bib-0027] Kay, K. M. (2006). Reproductive isolation between two closely related hummingbird‐pollinated neotropical gingers. Evolution, 60, 538–552. 16637499

[ece33147-bib-0028] Kirkpatrick, M. , & Ravigne, V. (2002). Speciation by natural and sexual selection: Models and experiments. American Naturalist, 159, S22–S35. 10.1086/338370 18707367

[ece33147-bib-0029] Kullenberg, B. (1951). *Ophrys insectifera* L et les insectes. Oikos, 3, 53–70.

[ece33147-bib-0030] Legendre, P. , & Gallagher, E. D. (2001). Ecologically meaningful transformations for ordination of species data. Oecologia, 129, 271–280. 28547606 10.1007/s004420100716

[ece33147-bib-0031] Legendre, P. , & Legendre, L. (1998). Numerical ecology. Amsterdam: Elsevier.

[ece33147-bib-0032] Linde, C. C. , Phillips, R. D. , Crisp, M. D. , & Peakall, R. (2014). Congruent species delineation of *Tulasnella* using multiple loci and methods. New Phytologist, 201, 6–12. 24028679 10.1111/nph.12492

[ece33147-bib-0033] Lowry, D. B. , Modliszewski, J. L. , Wright, G. A. , Wu, C. A. , & Willis, J. H. (2008). The strength and genetic basis of reproductive isolating barriers in flowering plants. Philosophical Transactions of the Royal Society B‐Biological Sciences, 363, 3009–3021. 10.1098/rstb.2008.0064 PMC2607309 18579478

[ece33147-bib-0034] Mant, J. , Peakall, R. , & Schiestl, F. P. (2005). Does selection on floral odor promote differentiation among populations and species of the sexually deceptive orchid genus *Ophrys*? Evolution, 59, 1449–1463. 16153031

[ece33147-bib-0035] Moyle, L. C. , Olson, M. S. , & Tiffin, P. (2004). Patterns of reproductive isolation in three angiosperm genera. Evolution, 58, 1195–1208. 15266970 10.1111/j.0014-3820.2004.tb01700.x

[ece33147-bib-0036] Okamoto, T. , Okuyama, Y. , Goto, R. , Tokoro, M. , & Kato, M. (2015). Parallel chemical switches underlying pollinator isolation in Asian Mitella. Journal of Evolutionary Biology, 28, 590–600. 25615872 10.1111/jeb.12591 PMC4418413

[ece33147-bib-0037] Oksanen, J. , Blanchet, F. G. , Kindt, R. , Legendre, P. , Minchin, P. R. , O'Hara, R. B. , Simpson, G. L. , Solymos, P. , & Stevens, M. H. H. (2012). Vegan: Community ecology package. Vienna: R Foundation for Statistical Computing.

[ece33147-bib-0038] Otero, J. T. , & Flanagan, N. S. (2006). Orchid diversity—Beyond deception. Trends in Ecology & Evolution, 21, 64–65. 16701475 10.1016/j.tree.2005.11.016

[ece33147-bib-0039] Peakall, R. (1990). Responses of male *Zaspilothynnus trilobatus* Turner wasps to females and the sexually deceptive orchid it pollinates. Functional Ecology, 4, 159–167.

[ece33147-bib-0040] Peakall, R. , Ebert, D. , Poldy, J. , Barrow, R. A. , Francke, W. , Bower, C. C. , & Schiestl, F. P. (2010). Pollinator specificity, floral odour chemistry and the phylogeny of Australian sexually deceptive *Chiloglottis* orchids: Implications for pollinator‐driven speciation. New Phytologist, 188, 437–450. 20561345 10.1111/j.1469-8137.2010.03308.x

[ece33147-bib-0041] Peakall, R. , & Whitehead, M. R. (2014). Floral odour chemistry defines species boundaries and underpins strong reproductive isolation in sexually deceptive orchids. Annals of Botany, 113, 341–355. 24052555 10.1093/aob/mct199 PMC3890385

[ece33147-bib-0042] Pecoraro, L. , Girlanda, M. , Liu, Z. J. , Huang, L. Q. , & Perotto, S. (2015). Molecular analysis of fungi associated with the Mediterranean orchid *Ophrys bertolonii* Mor. Annals of Microbiology, 65, 2001–2007.

[ece33147-bib-0043] Phillips, R. D. , Barrett, M. D. , Dalziell, E. L. , Dixon, K. W. , & Swarts, N. D. (2016). Geographical range and host breadth of *Sebacina* orchid mycorrhizal fungi associating with *Caladenia* in south‐western Australia. Botanical Journal of the Linnean Society, 182, 140–151.

[ece33147-bib-0044] Ramsey, J. , Bradshaw, H. D. , & Schemske, D. W. (2003). Components of reproductive isolation between the monkeyflowers *Mimulus lewisii* and *M‐cardinalis* (Phrymaceae). Evolution, 57, 1520–1534. 12940357 10.1111/j.0014-3820.2003.tb00360.x

[ece33147-bib-0045] Rieseberg, L. H. , & Willis, J. H. (2007). Plant speciation. Science, 317, 910–914. 17702935 10.1126/science.1137729 PMC2442920

[ece33147-bib-0046] Roche, S. A. , Carter, R. J. , Peakall, R. , Smith, L. M. , Whitehead, M. R. , & Linde, C. C. (2010). A narrow group of monophyletic *Tulasnella* (Tulasnellaceae) symbiont lineages are associated with multiple species of *Chiloglottis* (orchidaceae): Implications for orchid diversity. American Journal of Botany, 97, 1313–1327. 21616884 10.3732/ajb.1000049

[ece33147-bib-0047] Schatz, B. , Geoffroy, A. , Dainat, B. , Bessiere, J. M. , Buatois, B. , Hossaert‐McKey, M. , & Selosse, M. A. (2010). A case study of modified interactions with symbionts in a hybrid Mediterranean orchid. American Journal of Botany, 97, 1278–1288. 21616880 10.3732/ajb.0900303

[ece33147-bib-0048] Schemske, D. W. (2010). Adaptation and the origin of species. American Naturalist, 176, S4–S25. 10.1086/657060 21043779

[ece33147-bib-0049] Schiestl, F. P. , Ayasse, M. , Paulus, H. F. , Löfstedt, C. , Hansson, B. S. , Ibarra, F. , & Francke, W. (1999). Orchid pollination by sexual swindle. Nature, 399, 421–422.

[ece33147-bib-0050] Schiestl, F. P. , Ayasse, M. , Paulus, H. F. , Löfstedt, C. , Hansson, B. S. , Ibarra, F. , & Francke, W. (2000). Sex pheromone mimicry in the early spider orchid (*Ophrys sphegodes*): Patterns of hydrocarbons as the key mechanism for pollination by sexual deception. Journal of Comparative Physiology a‐Sensory Neural and Behavioral Physiology, 186, 567–574. 10.1007/s003590000112 10947239

[ece33147-bib-0051] Schiestl, F. P. , & Marion‐Poll, F. (2002). Detection of physiologically active flower volatiles using gas chromatography coupled with electroantennography. In Jackson J. F., & Linskens H. F., (Eds.), Analysis of taste and aroma (pp. 173‐198). Berlin: Springer.

[ece33147-bib-0052] Schiestl, F. P. , & Schlüter, P. M. (2009). Floral isolation, specialized pollination, and pollinator behavior in orchids. Annual Review of Entomology, 54, 425–446. 10.1146/annurev.ento.54.110807.090603 19067636

[ece33147-bib-0053] Schlüter, P. M. , & Schiestl, F. P. (2008). Molecular mechanisms of floral mimicry in orchids. Trends in Plant Science, 13, 228–235. 18424223 10.1016/j.tplants.2008.02.008

[ece33147-bib-0054] Scopece, G. , Cozzolino, S. , Johnson, S. D. , & Schiestl, F. P. (2010). Pollination efficiency and the evolution of specialized deceptive pollination systems. The American Naturalist, 175, 98–105. 10.1086/648555 19909087

[ece33147-bib-0055] Scopece, G. , Musacchio, A. , Widmer, A. , & Cozzolino, S. (2007). Patterns of reproductive isolation in Mediterranean deceptive orchids. Evolution, 61, 2623–2642. 17908246 10.1111/j.1558-5646.2007.00231.x

[ece33147-bib-0056] Scopece, G. , Widmer, A. , & Cozzolino, S. (2008). Evolution of postzygotic reproductive isolation in a guild of deceptive orchids. American Naturalist, 171, 315–326. 10.1086/527501 18198999

[ece33147-bib-0057] Sedeek, K. E. M. , Scopece, G. , Staedler, Y. M. , Schonenberger, J. , Cozzolino, S. , Schiestl, F. P. , & Schlüter, P. M. (2014). Genic rather than genome‐wide differences between sexually deceptive Ophrys orchids with different pollinators. Molecular Ecology, 23, 6192–6205. 25370335 10.1111/mec.12992

[ece33147-bib-0058] Sedeek, K. E. M. , Whittle, E. , Guthorl, D. , Grossniklaus, U. , Shanklin, J. , & Schlüter, P. M. (2016). Amino acid change in an orchid desaturase enables mimicry of the pollinator's sex pheromone. Current Biology, 26, 1505–1511. 27212404 10.1016/j.cub.2016.04.018

[ece33147-bib-0059] Selosse, M.‐A. , Dubois, M.‐P. , & Alvarez, N. (2009). Do Sebacinales commonly associate with plant roots as endophytes? Mycological Research, 113, 1062–1069. 19616625 10.1016/j.mycres.2009.07.004

[ece33147-bib-0060] Sun, M. , Schlüter, P. M. , Gross, K. , & Schiestl, F. P. (2015). Floral isolation is the major reproductive barrier between a pair of rewarding orchid sister species. Journal of Evolutionary Biology, 28, 117–129. 25382492 10.1111/jeb.12544

[ece33147-bib-0061] Tang, L. L. , Yu, Q. , Sun, J. F. , & Huang, S. Q. (2007). Floral traits and isolation of three sympatric Aquilegia species in the Qinling Mountains, China. Plant Systematics and Evolution, 267, 121–128.

[ece33147-bib-0161] Triponez, Y. , Arrigo, N. , Pellissier, L. , & Schatz, B. , & Alvarez, N. (2013). Morphological, ecological and genetic aspects associated with endemism in the Fly Orchid group. Molecular Ecology, 22, 1431–1446. 23331669 10.1111/mec.12169

[ece33147-bib-0062] Van der Niet, T. , Peakall, R. , & Johnson, S. D. (2014). Pollinator‐driven ecological speciation in plants: New evidence and future perspectives. Annals of Botany, 113, 199–211. 24418954 10.1093/aob/mct290 PMC3890394

[ece33147-bib-0063] Van der Niet, T. , Pirie, M. D. , Shuttleworth, A. , Johnson, S. D. , & Midgley, J. J. (2014). Do pollinator distributions underlie the evolution of pollination ecotypes in the Cape shrub *Erica plukenetii*? Annals of Botany, 113, 301–315. 24071499 10.1093/aob/mct193 PMC3890384

[ece33147-bib-0064] Vereecken, N. J. , Cozzolino, S. , & Schiestl, F. P. (2010). Hybrid floral scent novelty drives pollinator shift in sexually deceptive orchids. BMC Evolutionary Biology, 10, 1–12. 20409296 10.1186/1471-2148-10-103 PMC2875231

[ece33147-bib-0065] Waelti, M. O. , Muhlemann, J. K. , Widmer, A. , & Schiestl, F. P. (2008). Floral odour and reproductive isolation in two species of *Silene* . Journal of Evolutionary Biology, 21, 111–121. 18031491 10.1111/j.1420-9101.2007.01461.x

[ece33147-bib-0066] Waser, N. M. , & Campbell, D. R. (2004). Ecological speciation in flowering plants In Dieckmann U., Doebeli M., Metz M. J., & Tautz D. (Eds.), Adaptive speciation (pp. 264–277). Cambridge: Cambridge University Press.

[ece33147-bib-0067] Waterman, R. J. , & Bidartondo, M. I. (2008). Deception above, deception below: Linking pollination and mycorrhizal biology of orchids. Journal of Experimental Botany, 59, 1085–1096. 18316318 10.1093/jxb/erm366

[ece33147-bib-0068] Whitehead, M. R. , & Peakall, R. (2014). Pollinator specificity drives strong prepollination reprodutive isolation in sympatric sexually deceptive orchids. Evolution, 68, 1561–1575. 24527666 10.1111/evo.12382

[ece33147-bib-0069] Widmer, A. , Lexer, C. , & Cozzolino, S. (2009). Evolution of reproductive isolation in plants. Heredity, 102, 31–38. 18648386 10.1038/hdy.2008.69

[ece33147-bib-0070] Wong, B. B. M. , & Schiestl, F. P. (2002). How an orchid harms its pollinator. Proceedings of the Royal Society of London Series B‐Biological Sciences, 269, 1529–1532. 10.1098/rspb.2002.2052 PMC1691071 12184821

[ece33147-bib-0071] Xu, S. , Schlüter, P. M. , Grossniklaus, U. , & Schiestl, F. P. (2012). The genetic basis of pollinator adaptation in a sexually deceptive orchid. PLoS Genetics, 8, e1002889. 22916031 10.1371/journal.pgen.1002889 PMC3420943

[ece33147-bib-0072] Xu, S. , Schlüter, P. M. , & Schiestl, F. P. (2012). Pollinator‐driven speciation in sexually deceptive orchids. International Journal of Ecology, 2012, 9: 285081. doi:10.1155/2012/285081

[ece33147-bib-0073] Xu, S. , Schlüter, P. M. , Scopece, G. , Breitkopf, H. , Gross, K. , Cozzolino, S. , & Schiestl, F. P. (2011). Floral isolation is the main reproductive barrier among closely related sexually deceptive orchids. Evolution, 65, 2606–2620. 21884059 10.1111/j.1558-5646.2011.01323.x

