# Supplementary figures and images for: Floral scent and species divergence in a pair of sexually deceptive orchids
Source: Ecol Evol. 2017 Jun 28;7(15):6023–34. doi: 10.1002/ece3.3147 (PMC5551101; doi:10.1002/ece3.3147)

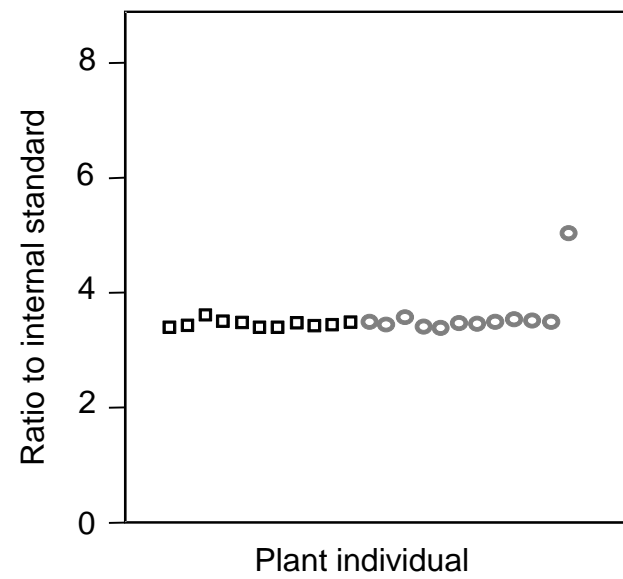

Supplement: Supplementary file 2 [file ECE3-7-6023-s002.pdf]

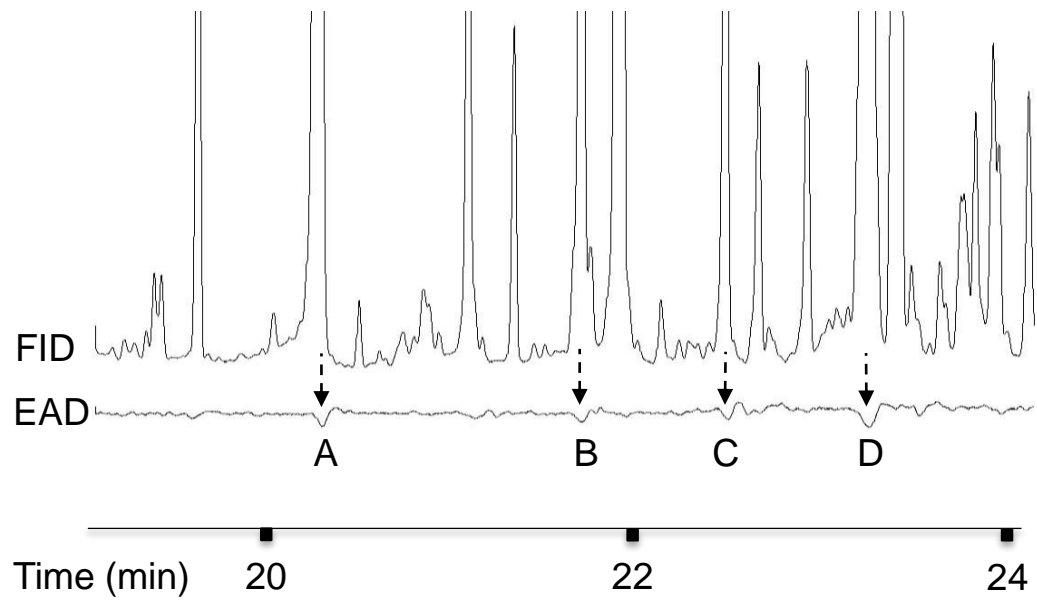

Supplement: Supplementary file 3 [file ECE3-7-6023-s003.pdf]

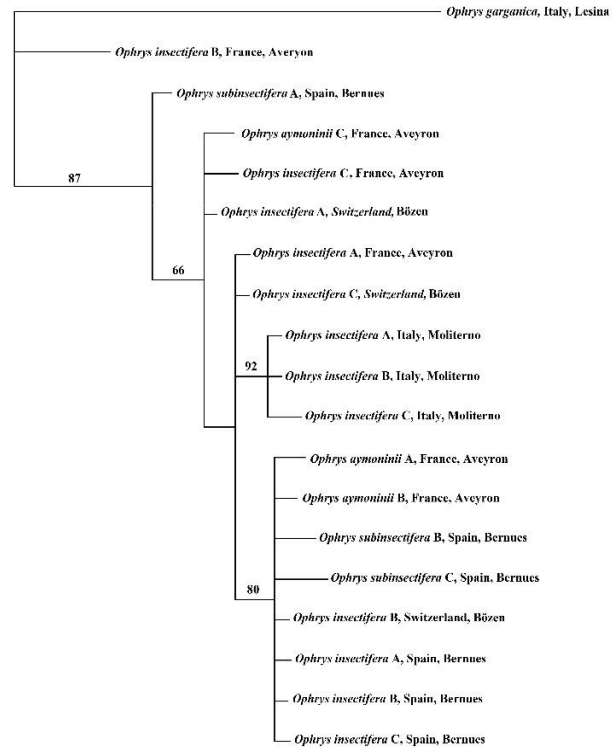

Supplement: Supplementary file 4 [file ECE3-7-6023-s004.pdf]
