# Supplementary material for: Floral scent and species divergence in a pair of sexually deceptive orchids
Source: Ecol Evol. 2017 Jun 28;7(15):6023–34. doi: 10.1002/ece3.3147 (PMC5551101; doi:10.1002/ece3.3147)
Supplement: Supplementary file 5 [file ECE3-7-6023-s005.docx]

**Fig. S1**: GPS coordinates (x-axis = longitude, y-axis = latitude) of three natural sympatric *O. insectifera* (grey circles) and *O. aymoninii* (black squares) populations in the Parc Naturel Régional des Grands-Causses in Aveyron, France. The figure shows the often interspersed occurrence of the two species.

**Fig. S2** Ploidy levels of pollinia from *O. insectifera* and *O. aymoninii plants*. Each data point represents the relative ratio between pollinia and internal standard from an individual plant (black squares= *O. aymoninii*, grey circles= *O. insectifera*).

**Fig. S3** Gas chromatographic analyses with electroantennographic detection (GC-EAD) of *Ophrys aymoninii* labellum extract, using antennae of a male *Andrena combinata* bees, the pollinator of *O. aymoninii.* Pollinators respond to four peaks identified as A = tricosane, B = (*Z*)-9-pentacosene, C = octyl palmitate, D = (*Z*)-9-heptacosene/nonyl palmitate. FID, flame-ionization detector.

**Fig. S4:**  Phylogenetic relationships interred from Bayesian analysis of three combined nuclear loci. Bayesian posterior probabilities (PP) are given above branches.

**Table S1 –** The 19 fungal OTUs found in *O. insectifera* and *O. aymoninii*, with number of sequences (Seq.) amplified among the 396 mycorrhizal samples and number of individual orchids (Ind.) where the OUT was found (out of, respectively, 26 and 24 sampled individuals).

|  | GB accession number(s) | Putative taxonomic affiliation | *O. aymoninii* | | *O. insectifera* | |
| --- | --- | --- | --- | --- | --- | --- |
|  |  |  | Seq. | Ind. | Seq. | Ind. |
| Orchid mycorrhizal fungi | |  | 91 | 27 | 73 | 30 |
| T1 | KF871201 | *Tulasnella* | 78 | 23 | 60 | 21 |
| T2 | KF871202 | *Tulasnella* | 3 | 1 | 1 | 1 |
| T3 | KF871203 | *Tulasnella* | 0 | 0 | 1 | 1 |
| S1 | KF871204 | Sebacinales cl. B | 2 | 1 | 11 | 7 |
| S2 | KF871205 | Sebacinales cl. B | 8 | 2 | 0 | 0 |
| Endophytic fungi | |  | 8 | 7 | 6 | 6 |
| E1 | KF871206 | *Leptodontidium sp.* | 0 | 0 | 3 | 3 |
| E2 | KF871207 | *Tetracladium sp.* | 2 | 2 | 1 | 1 |
| E3 | KF871208 | *Tetracladium sp.* | 1 | 1 | 0 | 0 |
| E4 | KF871209 | *Tetracladium sp.* | 0 | 0 | 1 | 1 |
| E5 | KF871210 | *Neonectria sp.* | 1 | 1 | 0 | 0 |
| E6 | KF871211 | *Gymnopus sp.* | 2 | 1 | 0 | 0 |
| E7 | KF871212 | Helotiales sp. | 0 | 0 | 1 | 1 |
| E8 | KF871213 | *Ceratobasidium sp.* | 1 | 1 | 0 | 0 |
| E9 | KF871214 | *Cladosporium sp.* | 1 | 1 | 0 | 0 |
| Ectomycorrhizal fungi | |  | 8 | 5 | 1 | 1 |
| M1 | KF871215 | *Rhizopogon sp.* | 5 | 2 | 0 | 0 |
| M2 | KF871216 | *Tricholoma sp.* | 1 | 1 | 0 | 0 |
| M3 | KF871217 | *Russula sp.* | 1 | 1 | 0 | 0 |
| M4 | KF871218 | Aethaliales sp. | 1 | 1 | 0 | 0 |
| M5 | KF871219 | Thelephoraceae sp. | 0 | 0 | 1 | 1 |

**Table S2**: Coordinates of all 7 populations with description of the surroundings

| **Population** | **Species** | **Coordinates** | **Description** |
| --- | --- | --- | --- |
| Avey 2 | *O. aymoninii* | 44° 1' 40'' N, 3° 15' 52'' E | Dense pine forest with open grass patches |
| Avey 3 | *O. aymoninii*  *O. insectifera* | 43° 59' 56'' N, 3° 13' 39'' E | Dense pine forest and open grass fields with single pines |
| Avey 4 | *O. aymoninii*  *O. insectifera* | 43° 47' 29'' N, 3° 27' 43'' E | Dense pine forest and open grass fields with single pines |
| Avey 5 | *O. insectifera* | 43° 59' 30'' N, 2° 56' 41'' E | Free grass field bordered by oak forest and wheat field |
| Avey 5_1 | *O. insectifera* | 44° 0' 18'' N, 2° 57' 36'' E | Pine forest with abandoned vineyard |
| Avey 6 | *O. aymoninii*  *O. insectifera* | 43° 57' 26'' N, 3° 4' 52'' E | Dense pine forest and open grass fields with single pines |
| Avey 8 | *O. insectifera* | 43° 54' 33'' N, 3° 17' 41'' E | Dense pine forest |
