# Supplementary material for: Floral scent and species divergence in a pair of sexually deceptive orchids
Source: Ecol Evol. 2017 Jun 28;7(15):6023–34. doi: 10.1002/ece3.3147 (PMC5551101; doi:10.1002/ece3.3147)
Supplement: Supplementary file 1 [file ECE3-7-6023-s001.pdf]

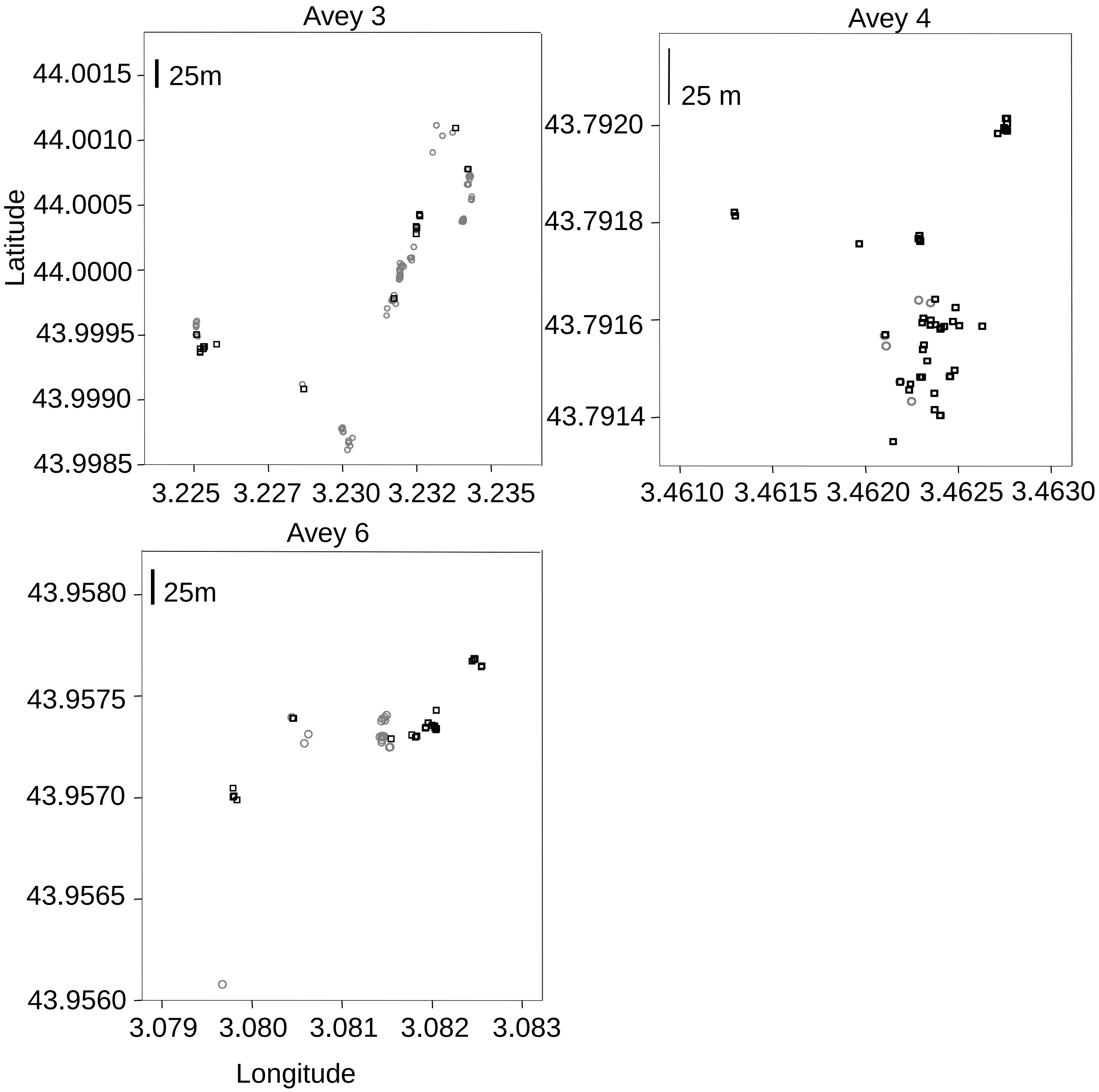
Avey 3
25m
44.0015
44.0010
44.0005
44.0000
43.9995
43.9990
43.9985
3.225
3.227
3.230
3.232
3.235
Latitude
Avey 4
25 m
43.7920
43.7918
43.7916
43.7914
3.4610
3.4615
3.4620
3.4625
3.4630
Avey 6
25m
43.9580
43.9575
43.9570
43.9565
43.9560
3.079
3.080
3.081
3.082
3.083
Longitude
